# Supplementary material for: Somatic cells compartmentalise their carbohydrate metabolism to sustain germ cell survival
Source: EMBO J. 2026 Jun 4;45(14):4910–30. doi: 10.1038/s44318-026-00815-y (PMC13373199; doi:10.1038/s44318-026-00815-y)
Supplement: Supplementary file 14 — Expanded View Figures [file 44318_2026_815_MOESM14_ESM.pdf]

Expanded View Figures

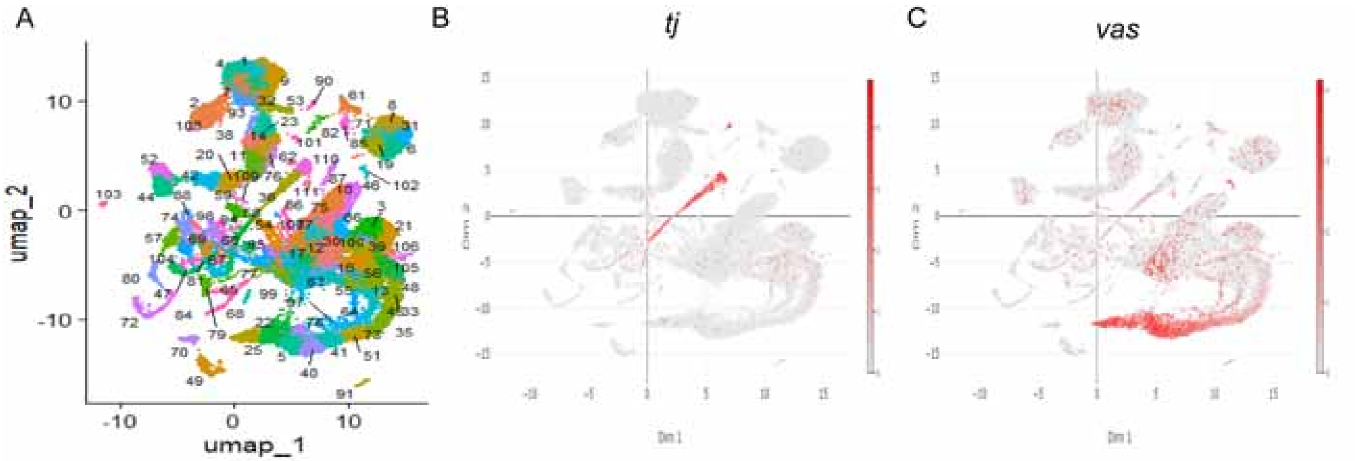

D

Comparison of somatic clusters 62, 36, 58 vs germ clusters 25 and 22										
GO term (FDR value)	Genes									
Biosynthesis of amino acids (FDR=0,003)	*Eno*	*Gapdh2*	*Pfk*	*Pgam1*	*Tpi*	*aay*	*Alat*	*Arg*	*Bcat*	*CG3483*
	CG32026	*Glts*	*Gs1*	*Gs2*	*Idh*	*Idh3g*	*Irp-1B*	*P5CS*	*Pcb*	*Phgdh*
	Psat	*Pykl2*	*Pykl5*	*Sams*	*Taldo*	*Tkt*	*Tktl*			
Carbon metabolism (FDR=0,003)	*Eno*	*Gapdh2*	*Hex-A*	*Pfk*	*Pgam1*	*Tpi*	*aay*	*Acat1*	*Acat2*	*AcCoAS*
	Alat	*CG3483*	*CG5577*	*CG17544*	*CG17896*	*CG32026*	*CG32487*	*CG32488*	*Fbp*	*G6pd*
	G6pdl	*Gdh*	*Hibadh*	*Idh*	*Idh3g*	*Irp-1B*	*Mdh1*	*Mdh2*	*Ogdh1*	*Pcb*
	Pgd	*Phgdh*	*Psat*	*Pykl2*	*Pykl5*	*SdhA*	*SdhAL*	*Taldo*	*Tkt*	*Tktl*

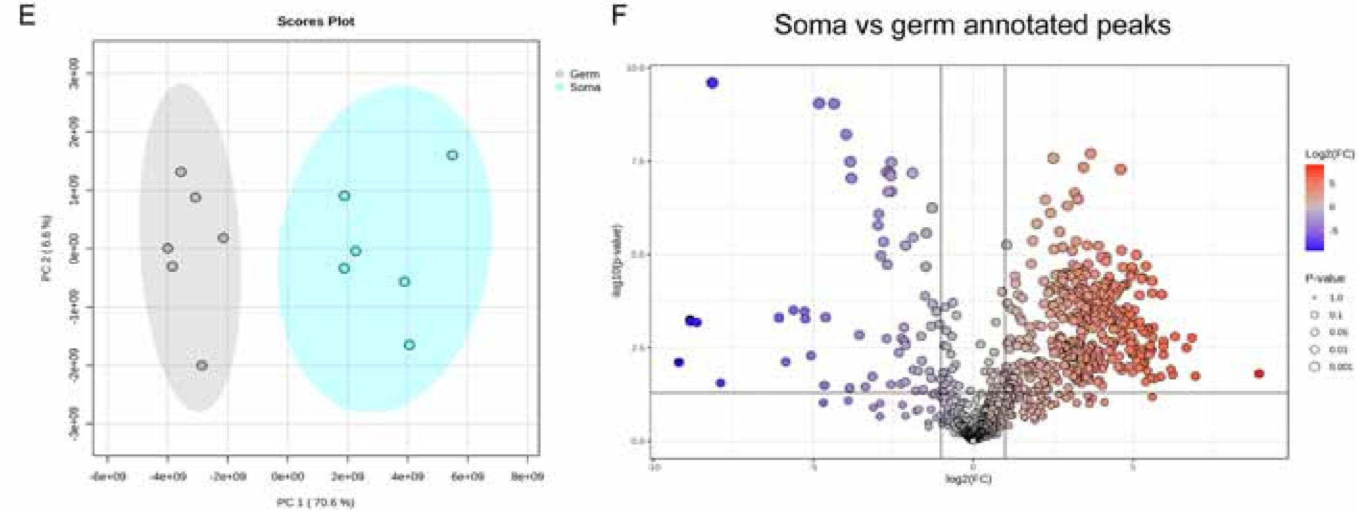

Figure EV1. Somatic and germ cells have different metabolic profiles.

(**A**) UMAP representation showing the clustering and annotation generated by Raz et al of the Fly Cell Atlas single-nucleus RNA sequencing of the testis. (**B**) Gene expression plot showing *tj* expression on the testis UMAP identifying clusters 62, 36 and 58 as corresponding to CySCs and early cyst cells. (**C**) Gene expression plot showing *vasa (vas)* expression on the testis UMAP representation, identifying clusters 25, 22 and 5 as corresponding to early stages of germ cell development. (**D**) Table listing the two most significantly enriched GO terms in genes enriched in early cyst cells (clusters 62, 36 and 58) compared to early germline clusters (25 and 22). The genes contained within the GO categories are shown, and those encoding glycolytic enzymes are highlighted in yellow. (**E**) Principal component analysis score plot from the mass spectrometry data from the independent replicates of sorted cyst (blue dots) and germ cells (grey dots). (**F**) Volcano plot showing the relative enrichment of all annotated mass spectrometry peaks in cyst cells compared to germ cells. *Y* axis shows *P* values as determined by *t* test. Data points represent mean values from 6 biological replicates for each condition. Source data are available online for this figure.

 © The Author(s)

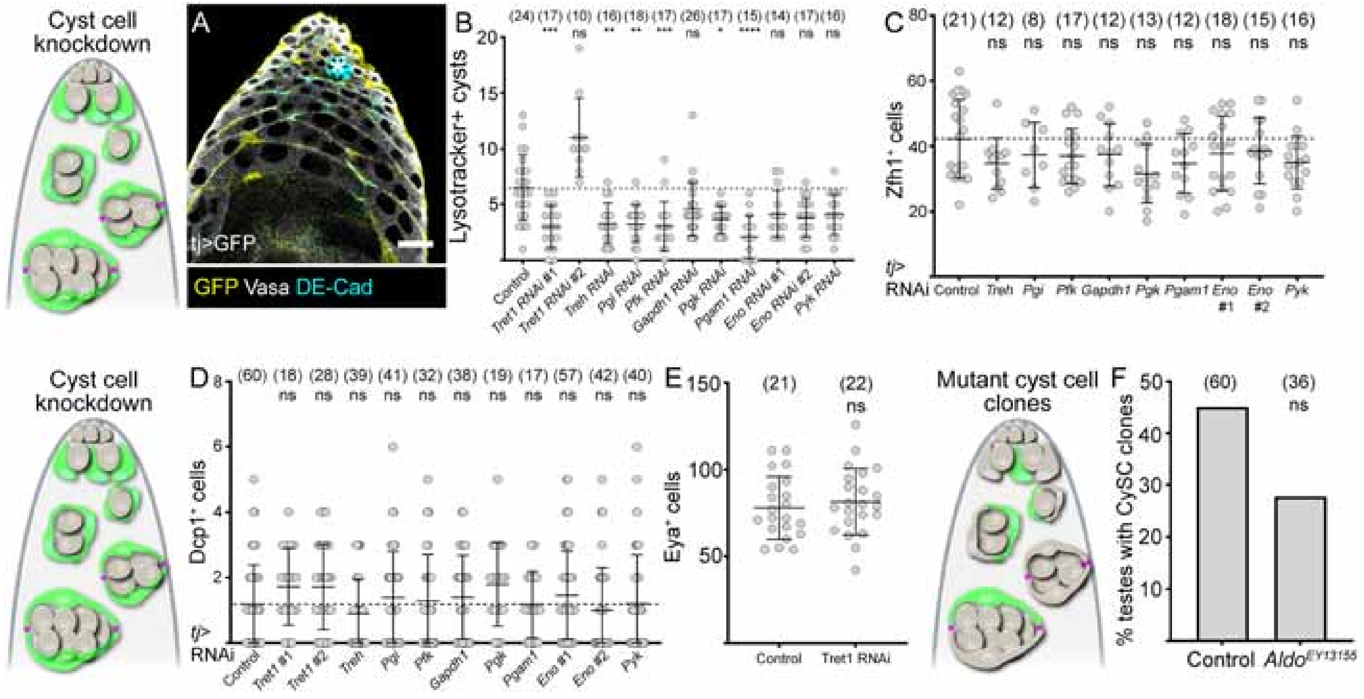

Figure EV2. Knockdown of genes encoding glycolytic enzymes does not affect CySC numbers or cyst cell survival.

(**A**) Confocal image of a testis expressing GFP (yellow) in somatic cells driven by *tj-Gal4*. Vasa (white) labels germ cells, DE-Cad (cyan) labels cell outlines. Scale bar: 20 µm. The diagram on the left represents a testis apical tip and highlights *tj-Gal4* expression in green. (**B**) Graph showing the number of Lysotracker-positive germ cell cysts in each of the parental strains used for glycolytic enzyme knockdown, compared to a wild-type strain (Oregon R). *Tret1* #1, $P = 0.001$; *Tret1* #2, $P = 0.502$; *Treh*, $P = 0.0037$; *Pgi*, $P = 0.0043$; *Pfk*, $P = 0.0002$; *Gapdh1*, $P = 0.3112$; *Pgk*, $P = 0.0399$; *Pgam1*, $P < 0.0001$; *Eno* #1, $P = 0.1591$; *Eno* #2, $P = 0.0787$; *Pyk*, $P = 0.2548$. ****$P < 0.0001$, ***$P < 0.001$, **$P < 0.01$, *$P < 0.05$, Kruskal–Wallis and Dunn's multiple comparisons tests. (**C**) Graph showing the number of Zfh1-positive, Eya-negative cells in control testes or testes in which the indicated genes encoding glycolytic enzymes were knocked down in cyst cells. Significance was assessed using Kruskal–Wallis and Dunn's multiple comparisons tests. (**D**) Graph showing the number of Dcp-1-positive cells in control testes or testes in which the indicated genes encoding glycolytic enzymes were knocked down in cyst cells. Significance was assessed using Kruskal–Wallis and Dunn's multiple comparisons tests. (**E**) Graph showing the number of Eya-positive cyst cells in control testes or testes in which *Tret1* was knocked down. Significance was assessed using a Mann–Whitney test. (**F**) Graph showing the recovery rate of control and *Aldo*^{EY13155} somatic clones. No significant difference was detected using Fisher's exact test. All graphs: *N* values are shown in brackets and refer to the number of testes analysed, error bars indicate standard deviation. Source data are available online for this figure.

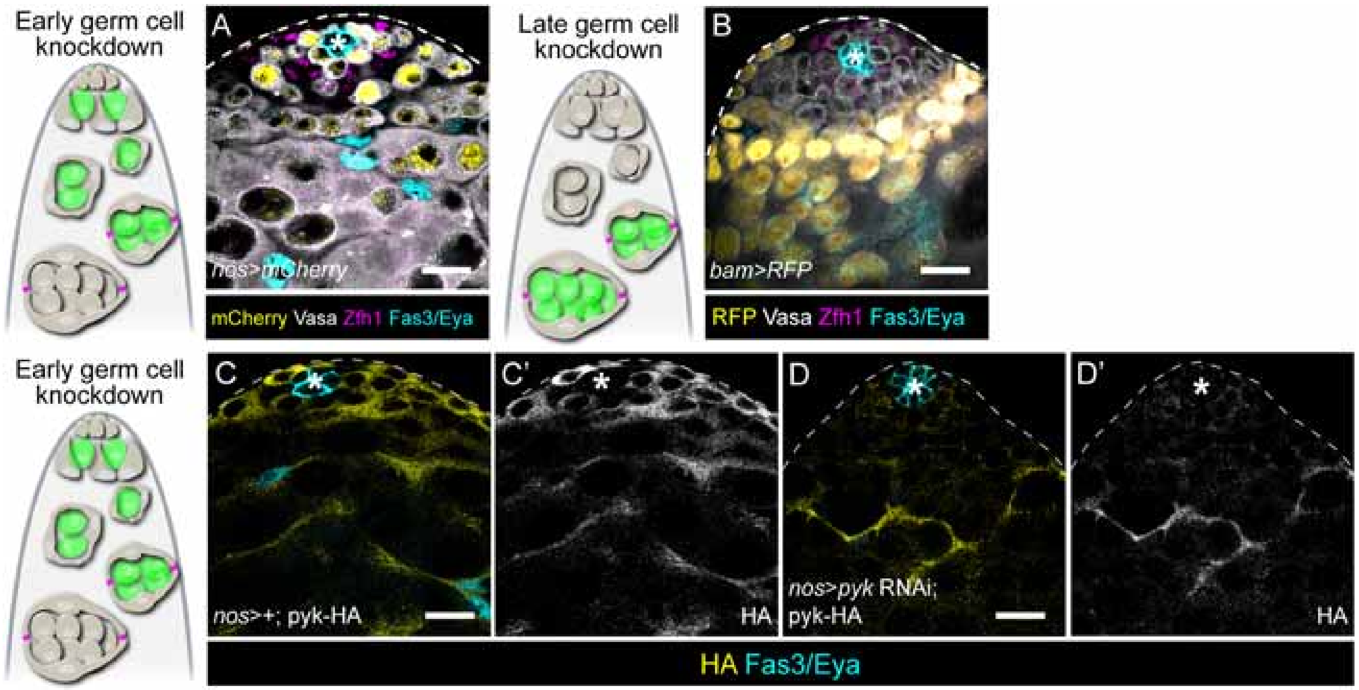

Figure EV3. Driver expression and RNAi knockdown in the germline.

(**A**, **B**) Confocal images of testes expressing mCherry (yellow) in early germ cells driven by *nos-Gal4* (**A**) or RFP (yellow) in differentiating germ cells driven by *bam-Gal4* (**B**). Vasa (white) labels germ cells, Zfh1 (magenta) labels CySCs, Fas3 and Eya (cyan) label the hub and cyst cells, respectively. Scale bar: 20 μm. The diagrams on the left represent a testis apical tip and highlight the domains of *nos-Gal4* and *bam-Gal4* expression in green. (**C**, **D**) Confocal images of testes from *Pyk-HA* control flies (**C**) or flies in which *Pyk* was knocked down in early germ cells with *nos-Gal4*, labelled with antibodies against HA (yellow, single channel (**C′**, **D′**)) and Fas3 and Eya (cyan) to label the hub and cyst cells, respectively. All panels: the testis is outlined with a dashed line, and asterisks indicate the hub. Scale bars: 20 μm. Source data are available online for this figure.

 © The Author(s)

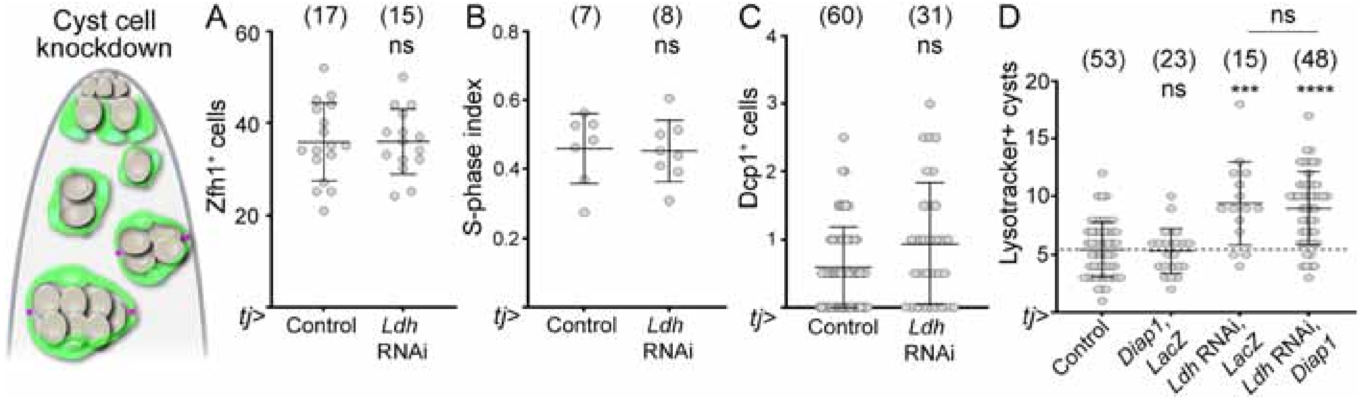

Figure EV4. *Ldh* is not required autonomously in CySCs or cyst cells.

(**A**) Graph showing the number of Zfh1-positive, Eya-negative cells in control testes or testes in which *Ldh* was knocked down in cyst cells. Significance was assessed using a Mann–Whitney test. (**B**) S-phase index of Zfh1-positive CySCs in control testes and testes in which *Ldh* was knocked down in cyst cells. Significance was assessed using a Student's *t* test. (**C**) Graph showing the number of Dcp-1-positive cells in control testes and testes in which *Ldh* was knocked down in cyst cells. Significance was assessed using a Mann–Whitney test. (**D**) Graph showing the number of Lysotracker-positive germ cell cysts in control testes and testes in which *Ldh* was knocked down in cyst cells, with or without overexpression of the apoptosis inhibitor Diap1. *Diap1; LacZ* vs control: $P > 0.999$; *Ldh* RNAi; *LacZ* vs control: $P = 0.0006$; *Ldh* RNAi; *Diap1* vs control: $P < 0.0001$; *Ldh* RNAi; *LacZ* vs *Ldh* RNAi; *Diap1*: $P > 0.9999$, determined by Kruskal–Wallis and Dunn's multiple comparisons tests. All graphs: *N* values are shown in brackets and refer to the number of testes analysed, error bars indicate standard deviation. Source data are available online for this figure.

© The Author(s)

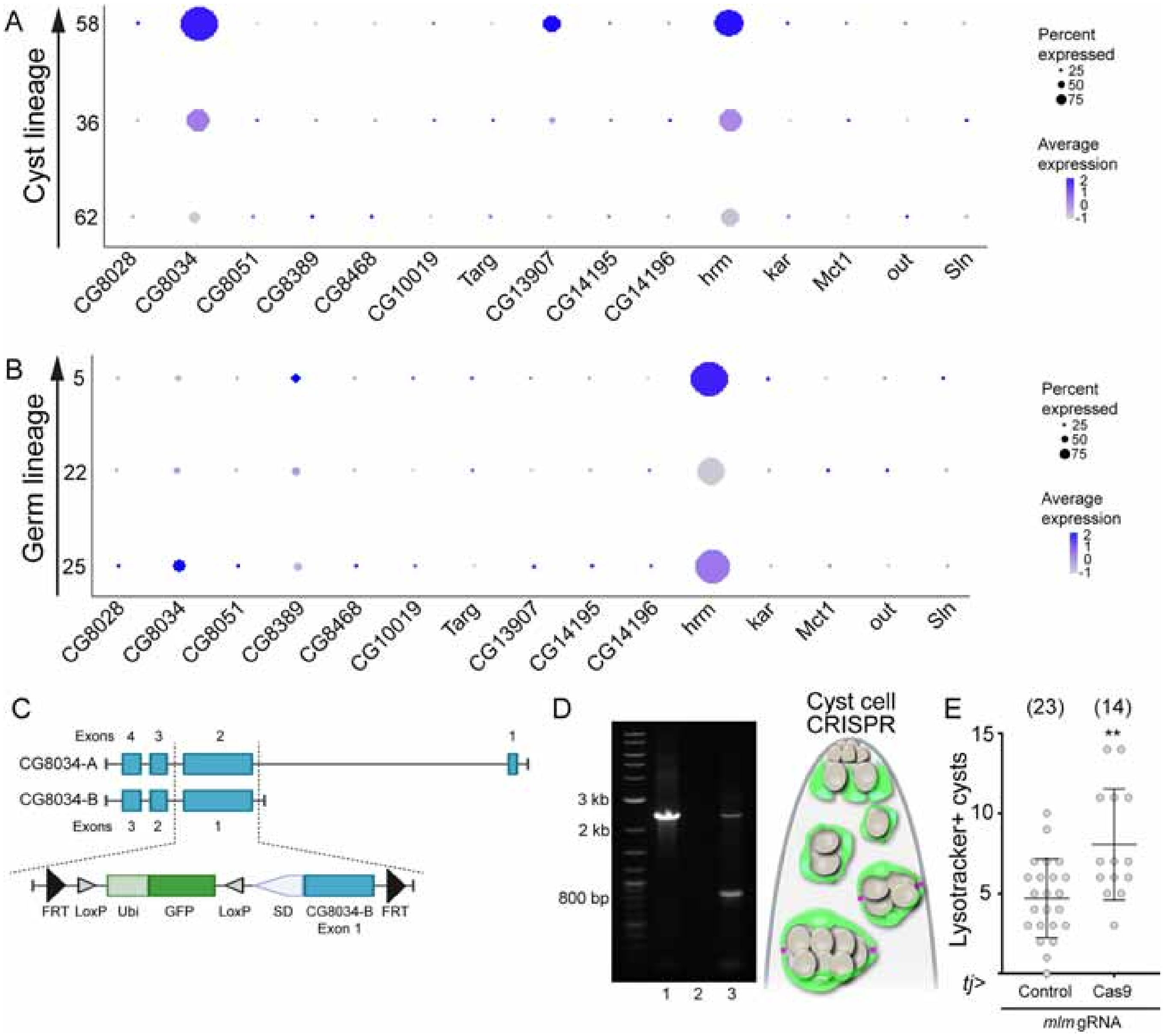

Figure EV5. The monocarboxylate transporter encoded by *CG8034/mlm* is required for germ cell survival.

(**A**) Dot plot showing average expression from the Fly Cell Atlas dataset of the indicated genes encoding MCTs in the cell clusters corresponding to CySCs (62) and early cyst cells (36 and 58). (**B**) Dot plot showing average expression from the Fly Cell Atlas dataset of the indicated genes encoding MCTs in the cell clusters corresponding to early (25), and late spermatogonia (22) and cells at the spermatogonia-spermatocyte transition (5). The size of the dots indicates the percentage of expressing cells in each cluster, and the colour represents the expression level. Note that *chaski* was not identified in this dataset. (**C**) Diagram showing the *CG8034/mlm* locus and two predicted transcripts, with the location of the CRISPR deletion indicated by dotted lines. This region was replaced by a cassette restoring the deleted exon, flanked by FRT sites to enable conditional excision. (**D**) Image of a DNA gel following PCR amplification of the genomic DNA using primers located in the homology arms of the inserted cassette. Lane 1 corresponds to a control, giving the expected size of 2.4 kb. In a homozygous mutant (lane 2), the expected size is 6.3kb, but this band was not obtained. In lane 3, PCR of DNA from a heterozygous larva carrying *hs-Flp* that was subjected to heat shocks shows the wild-type band and a 0.8 kb band corresponding to the expected size after excision of the exon. (**E**) Graph showing the number of Lysotracker-positive germ cell cysts in testes from animals expressing gRNAs targeting *mlm* with and without expression of Cas9 in cyst cells with *tj-Gal4*. $P = 0.0031$, determined by Kruskal–Wallis and Dunn's multiple comparisons tests. *N* values are shown in brackets and refer to the number of testes analysed; error bars indicate standard deviation. Source data are available online for this figure.

 © The Author(s)

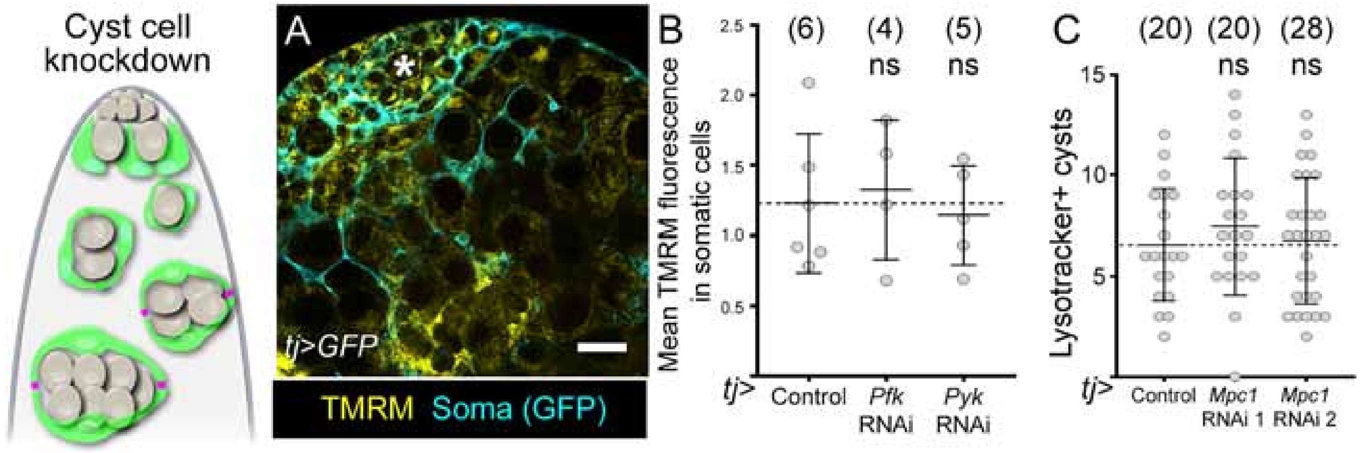

Figure EV6. Mitochondrial activity in cyst cells does not depend on autonomous pyruvate consumption.

(**A**) Airyscan image of a testis in which GFP (cyan) was expressed with *tj*-Gal4 to identify cyst cells and labelled with the mitochondrial membrane potential-sensitive dye TMRM (yellow). The hub is indicated with an asterisk. Scale bar: 20 μm. (**B**) Graph showing TMRM mean intensity in cyst cells in control testes and testes in which *Pfk* or *Pyk* were knocked down. Significance was determined by Kruskal– Wallis and Dunn's multiple comparisons tests. (**C**) Graph showing the number of Lysotracker-positive cysts in control testes and testes in which *Mpc1* was knocked down in cyst cells. Significance was determined by Kruskal–Wallis and Dunn's multiple comparisons tests. All graphs: *N* values are shown in brackets and refer to the number of testes analysed. Source data are available online for this figure.

© The Author(s)
