# Supplementary material for: Somatic cells compartmentalise their carbohydrate metabolism to sustain germ cell survival
Source: EMBO J. 2026 Jun 4;45(14):4910–30. doi: 10.1038/s44318-026-00815-y (PMC13373199; doi:10.1038/s44318-026-00815-y)
Supplement: Supplementary file 2 — Peer Review File [file 44318_2026_815_MOESM2_ESM.pdf]

THE EMBO JOURNAL

Somatic cells compartmentalise their carbohydrate metabolism to sustain germ cell survival

Diego Sainz de la Maza, Holly Jefferson, Celine Brucker, Sonia Paoli, and Marc Amoyel

Corresponding author(s): Marc Amoyel (marc.amoyel@ucl.ac.uk), Diego Sainz de la Maza (diego.sainzdelamaza@ucl.ac.uk)

Review Timeline:

Submission Date:	10th Sep 25
Editorial Decision:	7th Oct 25
Revision Received:	1st Apr 26
Editorial Decision:	22nd Apr 26
Revision Received:	24th Apr 26
Accepted:	30th Apr 26

Editor: Ieva Gailite

Transaction Report:

(Note: With the exception of the correction of typographical or spelling errors that could be a source of ambiguity, letters and reports are not edited. Depending on transfer agreements, referee reports obtained elsewhere may or may not be included in this compilation. Referee reports are anonymous unless the Referee chooses to sign their reports.)

Dear Marc,

Thank you for submitting your manuscript for consideration by the EMBO Journal. We have now received comments from three reviewers, which are included below for your information.

As you will see, all reviewers are positive to enthusiastic in their assessment and appreciate the contribution of the study to the research field, while also indicating a number of generally reasonable concerns that would need to be addressed in the revised version. Based on the positive evaluation by our reviewers, I would like to invite you to address their comments in a revised manuscript. I think that it could be useful to discuss the revision, and in particular the feasibility of addressing the comments by reviewer #2, in more detail via email or phone/videoconferencing - please let me know which option you prefer.

We generally allow three months as standard revision time, which can be extended to six months in the case of major revisions. Should you foresee a problem in meeting this deadline, please let us know in advance to discuss an extension.

As a matter of policy, competing manuscripts published during this period will not negatively impact on our assessment of the conceptual advance presented by your study. However, please contact me as soon as possible upon publication of any related work to discuss the appropriate course of action.

When preparing your letter of response to the referees' comments, please bear in mind that this will form part of the Review Process File and will therefore be available online to the community. For more details on our Transparent Editorial Process, please visit our website: https://www.embopress.org/page/journal/14602075/authorguide#transparentprocess. Please also see the attached instructions for further guidelines on preparation of the revised manuscript.

Please feel free to contact me if have any further questions regarding the revision. Thank you for the opportunity to consider your work for publication, and I look forward to discussing your revision with you.

With best wishes,

Ieva

Ieva Gailite, PhD
Senior Scientific Editor
The EMBO Journal
Meyerhofstrasse 1
D-69117 Heidelberg
Tel: +4962218891309
i.gailite@embojournal.org

Instructions for preparing your revised manuscript:

Please make sure you upload a letter of response to the referees' comments together with the revised manuscript.

Please also check that the title and abstract of the manuscript are brief, yet explicit, even to non-specialists.

When assembling figures, please refer to our figure preparation guideline in order to ensure proper formatting and readability in print as well as on screen:
https://bit.ly/EMBOPressFigurePreparationGuideline
See also guidelines for figure legends: https://www.embopress.org/page/journal/14602075/authorguide#figureformat

At EMBO Press we ask authors to provide source data for the main manuscript figures. You will receive a separate email with instructions for providing source data with your revised manuscript, including how to upload and organize the files.

IMPORTANT: When you send the revision we will require
- a point-by-point response to the referees' comments, with a detailed description of the changes made (as a word file).
- a word file of the manuscript text.
- individual production quality figure files (one file per figure)
- a complete author checklist, which you can download from our author guidelines (https://www.embopress.org/page/journal/14602075/authorguide).
- Expanded View files (replacing Supplementary Information)
Please see out instructions to authors

https://www.embopress.org/page/journal/14602075/authorguide#expandedview
- a Reagents and Tools Table as part of the Methods section, which can be downloaded from our author guidelines (https://www.embopress.org/page/journal/14602075/authorguide#structuredmethods)

Please remember: Digital image enhancement is acceptable practice, as long as it accurately represents the original data and conforms to community standards. If a figure has been subjected to significant electronic manipulation, this must be noted in the figure legend or in the 'Materials and Methods' section. The editors reserve the right to request original versions of figures and the original images that were used to assemble the figure.

Further information is available in our Guide For Authors: https://www.embopress.org/page/journal/14602075/authorguide

We realize that it is difficult to revise to a specific deadline. In the interest of protecting the conceptual advance provided by the work, we recommend a revision within 3 months (5th Jan 2026). Please discuss the revision progress ahead of this time with the editor if you require more time to complete the revisions. Use the link below to submit your revision:

https://emboj.msubmit.net/cgi-bin/main.plex

Referee #1:

This study demonstrates the metabolic division of labor between male germline cells and their surrounding somatic cyst cells. The study provides compelling evidence for a model that cyst cells import trehalose, metabolize it into lactate which is transported into germ cells. Lactate is transported by a previous uncharacterized monocarboxylate transporter, named Milkman. Feeding the germ cells by lactate is necessary for their survival. In germ cells, lactate is converted to pyruvate, but its downstream metabolism is not studied further (an interesting topic for a follow-up study). The experimentation is mostly based of cell type specific loss-of-function genetics and fluorescent metabolite sensor as well as lysotracker based detection of germ cell death. This experimental setup allows dissecting the shared metabolism between the two cell types exceptionally robustly. This study will be of high interest for scientists working on germline cells, stem cells and spatial aspects of tissue metabolism.
The evidence provided strongly supports the main conclusions and the model.

All my concerns can be addressed in writing or data presentation:

1. The introduction is somewhat superficial and it does not properly introduce the state of the art in germline metabolism and the metabolic division of labor between germ cells and their neighboring somatic cells. Even though the discussion relates the finding with published work, a bit more context would be helpful already in the introduction to help the reader to understand and value the advance.

2. Figure 1 is hard to understand for someone, who is not familiar with the organization of the Drosophila testis or the cell types and their abbreviations. It would be helpful to add the information regarding marker expression into Figure 1A, in addition to the legend. With the current panels 1C-E it is difficult to compare the expression pattern of Tret1 vs Aldo and PyK, since the markers are different and the expression of the GFP reporter is nuclear in the case of Tret1. The picture gets further confused by mentioning Vasa positive cells in the body text of results, but not showing Vasa staining in the Figure. Please clarify these issues and make the figures more self-explanatory (to reduce the need to jump back and forth between the figure and legend) and help the reader to understand, are they expressed in the same or different cells. If different, explain what are the implications to the model proposed.

3. Figure 2A vs. B: why Vasa intensity is so different between the panels?

4. In Figure 3F-H the visible pyronic and lactonic sensor signal comes only from the tip (cyst cells or progenitors?), while the Aldo and PyK are strongly expressed in later stages (below the tip). Is this a real difference or due to technical reasons?

5.The authors show that mitochondrial activity in cyst cells is not affected by glycolysis (Figure S6), but do not comment on mitochondrial activity in germ cells and the influence of cyst cell glycolysis and/or lactate transport on it. Please elaborate.

6. A technical note regarding manuscript preparation for review: It helps the reviewer's job tremendously, if figure legends are placed directly under figures. Line numbering helps commenting the text.

Referee #2:

The manuscript by Diego Sainz de la Maza and colleagues investigates the metabolic coupling between differentiating germline

cells and their supporting somatic lineage in the Drosophila male testis. The authors present three main conclusions:

-1/ The glycolytic pathway is specifically upregulated in differentiated somatic cells, but not in differentiated germline cells.
-2/ Somatic glycolytic activity non-autonomously supports the survival of differentiated germ cells.
-3/ Metabolic compartmentalisation culminates in the exchange of lactate between somatic and germline cells, mediated by a newly characterised monocarboxylate transporter, CG8034.

These findings represent a significant contribution to our understanding of the metabolic regulation of male germline differentiation. The study provides valuable and novel data that merit publication. Nonetheless, several of the central conclusions would benefit from additional experimental support. Below, I outline key experiments that could help to strengthen the manuscript.

Major comments

-Related to Figure 2: While knockdown of glycolytic enzymes in cyst cells increases germline cell death, the same manipulation within germ cells themselves has no effect. On this basis, the authors propose a model of metabolic compartmentalisation. However, the observed phenotype is relatively mild: germline cell death increases by less than twofold compared with controls. The authors do not provide evidence that these cellular effects influence male fertility or reproductive capacity, and it is plausible that they do not. In this context, one could instead postulate redundancy between somatic and germline metabolic activities, ensuring robustness of a system essential for male fitness. To rule out this possibility, it would be important to knock down glycolytic enzymes simultaneously in both lineages (using tj and bam drivers together). If the resulting phenotype proves stronger than that observed with tj alone, this would significantly alter the conclusions, revealing a layer of redundancy rather than metabolic compartmentalisation alone.

-Related to Figure 4: The identification of CG8034 as the monocarboxylate transporter responsible for lactate transport is currently supported by a single RNAi line, which is insufficient. Additional experiments could include: (1) generating a clean CRISPR mutant, which is relatively straightforward; (2) performing a CRISPR-mediated somatic knockout using a gRNA line available from a stock center; and (3) conducting a rescue experiment by overexpressing an RNAi- or gRNA-resistant, codon-optimised form of CG8034.

-Related to Figure 5: In this final figure, the authors use genetic manipulations of Pdk and Pdha1 in cyst cells to modulate oxidative phosphorylation and the pool of pyruvate/lactate available for germ cells, thereby demonstrating metabolic compartmentalisation between somatic and germ cells. These experiments are very interesting but would be strengthened by rescue experiments. Specifically, the increased cell death observed upon Pdk knockdown should be rescued by simultaneous Mpc1 knockdown (as Mpc1 knockdown alone has no phenotype). Similarly, the decreased cell death observed upon Pdha1 knockdown should also be rescued by concurrent Mpc1 knockdown.

Minor comments
-Related to Figure 2: The levels of germline cell death appear inconsistent across panels C, G and H. In panel C, the control condition shows around five dying cysts per testis (increasing to 7-8 under experimental conditions), whereas in panels G and H the control condition already reaches 7-8 cysts and no further effect is observed in the experimental conditions. These discrepancies may stem from differences in genetic background. However, since the apparent phenotype overlaps with the control condition in some panels, additional controls are needed to substantiate the findings. At present, only a single control condition is shown. Both parental controls-Gal4 alone and RNAi alone-should be included in all three graphs, and it should be clearly indicated which control is displayed.

-Related to Figure 2: Although not essential, it would be informative to test whether sugar starvation exacerbates the phenotype. Showing an increased dependence of the germline on somatic glycolytic activity under nutrient stress could further strengthen the paper.

-Related to Figure 2: To rule out an indirect effect mediated by somatic cell death, the author quantifies Zfh1-positive cells. Zfh1 marks CySCs and their immediate daughters, while Eya labels post-mitotic, differentiated cyst cells. In this context, it would also be important to quantify Eya-positive cells in the Tret1 knockdown, for example, to confirm that terminal differentiation of the somatic lineage is not impaired

Referee #3:

This manuscript from the Amoyel lab asks a fundamentally important question about how somatic cells provide nutrients to germ cells, which are isolated through encapsulation by somatic cells. This is an important question that everyone should have wondered, but nobody has. By a series of elegant and convincing experiments, the paper convincingly demonstrates how somatic cells provide the product of glycolysis to the germ cells. Studying metabolism in a manner that maintains cell-type-specific resolution is extremely difficult. But this study achieved this at an unprecedented level (using imaging and genetics), and

contains 3 papers-worth conclusions. This is a fundamentally important paper, and I enthusiastically support publication of this paper.

Minor comments:
-Fig2: when glycolysis enzymes are inhibited in somatic cells, germ cell death was upregulated without changing somatic cell composition. But this is a very important point (germ cell death is not due to death of somatic cells), so it would be great if they can provide somewhat more quantitative data to support this point.

-they convincingly show that glycolytic enzymes (e.g. pyk) are not required in germline. But any speculation why it is expressed in germline if not necessary? (no need to even include in the revised text, if it is disruptive to the flow).

We thank the reviewers for their feedback and positive assessment of our manuscript. We have addressed the comments they raised through new experiments and additions to the text. In particular, we have added new figure panels in which we characterise a mutant allele of *CG8034/mlm*, which significantly strengthens our conclusions as to the role of this previously uncharacterised gene. Overall, we believe the manuscript is much improved and we hope the reviewers will now find it suitable for publication.

Referee #1:

This study demonstrates the metabolic division of labor between male germline cells and their surrounding somatic cyst cells. The study provides compelling evidence for a model that cyst cells import trehalose, metabolize it into lactate which is transported into germ cells. Lactate is transported by a previous uncharacterized monocarboxylate transporter, named Milkman. Feeding the germ cells by lactate is necessary for their survival. In germ cells, lactate is converted to pyruvate, but its downstream metabolism is not studied further (an interesting topic for a follow-up study). The experimentation is mostly based of cell type specific loss-of-function genetics and fluorescent metabolite sensor as well as lysotracker based detection of germ cell death. This experimental setup allows dissecting the shared metabolism between the two cell types exceptionally robustly. This study will be of high interest for scientists working on germline cells, stem cells and spatial aspects of tissue metabolism.
The evidence provided strongly supports the main conclusions and the model.

All my concerns can be addressed in writing or data presentation:

1. The introduction is somewhat superficial and it does not properly introduce the state of the art in germline metabolism and the metabolic division of labor between germ cells and their neighboring somatic cells. Even though the discussion relates the finding with published work, a bit more context would be helpful already in the introduction to help the reader to understand and value the advance.
We agree with the reviewer and thank them for the opportunity to improve our manuscript. In trying to keep the introduction short in our initial draft we omitted a lot of useful information. The introduction has been revised and is now longer and more comprehensive, and we hope provides more context for our work. We have introduced the *in vitro* mammalian work suggesting the existence of a lactate shuttle, and discussed in more detail the current state of knowledge on the role of somatic cells in the Drosophila testis. We note however that there has been no study to date indicating what support is provided by the cyst cells to the developing germline, an important gap that our study will begin to address.

2. Figure 1 is hard to understand for someone, who is not familiar with the organization of the Drosophila testis or the cell types and their abbreviations. It would be helpful to add the information regarding marker expression into Figure 1A, in addition to the legend. With the current panels 1C-E it is difficult to compare the expression pattern of Tret1 vs Aldo and PyK, since the markers are different and the expression of the GFP reporter is nuclear in the case of Tret1. The picture gets further confused by mentioning Vasa positive cells in the body text of results, but not showing Vasa staining in the Figure. Please clarify these issues and make the figures more self-explanatory (to reduce the need to jump back and forth between the figure and legend) and help the reader to understand, are they expressed in the same or different cells. If different, explain what are the implications to the model proposed.
We thank the reviewer for pointing this out and apologise for any confusion. We have now

repeated the stainings in Fig. 1 to use the same markers and make comparisons easier for readers. We have further added single channel images (Fig. 1C', D', E') to show the Tret1, Aldo and Pyk reporters more clearly as the Vasa channel can obscure other labels.

3. Figure 2A vs. B: why Vasa intensity is so different between the panels?
This was simply due to variability in the staining as in our hands, the quality of image obtained using the anti-Vasa antibody can depend on antibody batches and sample processing. While the aim of using the Vasa antibody was simply to show the presence of the cell types, we agree that the variation in intensity distracted from the fact that there is no detectable difference other than an increase in Lysotracker-positive cysts. We have now replaced the image in Fig. 2B with one that shows similar staining intensity as the control.

4. In Figure 3F-H the visible pyronic and lactonic sensor signal comes only from the tip (cyst cells or progenitors?), while the Aldo and PyK are strongly expressed in later stages (below the tip). Is this a real difference or due to technical reasons?
The pyronicSF signal in Fig. 3F-H is indeed higher at the apical tip of the testis. We consistently observe a pattern as shown in the figure, where high fluorescence is visible in Zfh1-positive cyst stem cells and cyst cells that are adjacent to this apical zone, and lower/undetectable levels in cyst cells further away from the hub that are further along the differentiation pathway. We used *tj-Gal4* to drive expression of the reporter, and as shown in Fig. S2, when we express GFP using the same driver, fluorescence is visible in cyst cells throughout the testis. Similarly, the CanlonicSF reporter (Fig. 3B) shows more even fluorescence among cyst stem cells and differentiating cyst cells. Thus, the pattern we observe is unlikely to be a technical problem with the driver. We note that when we knock down Lactate dehydrogenase (Fig. 3H), PyronicSF fluorescence becomes visible in cyst cells more distal to the hub, indicating that the reporter is expressed there but that in controls there is low fluorescence of the reporter. It is possible that this pattern reflects a lower dynamic range of reporter sensitivity than for CanlonicSF, but in any case it strongly suggests that pyruvate levels are lower in later differentiated cyst cells than in stem cells and early differentiated cells. This is not consistent with the increase in glycolytic gene expression we observe in differentiating cyst cells (Fig. 1C-F). One possible explanation for this is that we observe an upregulation of *lactate dehydrogenase (ldh)* in cyst cells as they differentiate. Given the high affinity of Ldh for pyruvate, it is possible that simply increasing Ldh expression is sufficient to deplete pyruvate levels. In sum, it is fairly difficult to conclude from these fixed samples whether the changes we observe whether a change in fluorescence reflects decreased production or increased flux. Our experimental data would argue for the latter. We have added some text in the results on page 6 (l.213-7) to describe this observation and its likely cause.

5.The authors show that mitochondrial activity in cyst cells is not affected by glycolysis (Figure S6), but do not comment on mitochondrial activity in germ cells and the influence of cyst cell glycolysis and/or lactate transport on it. Please elaborate.
This is indeed an important question and the role of lactate in germ cell metabolism is an area of active investigation in our lab. We are currently exploring several hypotheses including that lactate could act to sustain germ cell mitochondrial activity. However, this work is still at early stages, and we chose to focus this manuscript on demonstrating shuttling of lactate from the soma to the germline and how the soma sustains this. We feel that the cell autonomous requirements within germ cells would be better explored in a separate manuscript.

6. A technical note regarding manuscript preparation for review: It helps the reviewer's job

tremendously, if figure legends are placed directly under figures. Line numbering helps commenting the text.
We agree with the reviewer and apologise for the inconvenience. While having to upload image files separately makes it difficult to include the legend with the figure, we have added line numbers.

Referee #2:

The manuscript by Diego Sainz de la Maza and colleagues investigates the metabolic coupling between differentiating germline cells and their supporting somatic lineage in the Drosophila male testis. The authors present three main conclusions:

-1/ The glycolytic pathway is specifically upregulated in differentiated somatic cells, but not in differentiated germline cells.
-2/ Somatic glycolytic activity non-autonomously supports the survival of differentiated germ cells.
-3/ Metabolic compartmentalisation culminates in the exchange of lactate between somatic and germline cells, mediated by a newly characterised monocarboxylate transporter, CG8034.

These findings represent a significant contribution to our understanding of the metabolic regulation of male germline differentiation. The study provides valuable and novel data that merit publication. Nonetheless, several of the central conclusions would benefit from additional experimental support. Below, I outline key experiments that could help to strengthen the manuscript.

Major comments

-Related to Figure 2: While knockdown of glycolytic enzymes in cyst cells increases germline cell death, the same manipulation within germ cells themselves has no effect. On this basis, the authors propose a model of metabolic compartmentalisation. However, the observed phenotype is relatively mild: germline cell death increases by less than twofold compared with controls. The authors do not provide evidence that these cellular effects influence male fertility or reproductive capacity, and it is plausible that they do not. In this context, one could instead postulate redundancy between somatic and germline metabolic activities, ensuring robustness of a system essential for male fitness. To rule out this possibility, it would be important to knock down glycolytic enzymes simultaneously in both lineages (using tj and bam drivers together). If the resulting phenotype proves stronger than that observed with tj alone, this would significantly alter the conclusions, revealing a layer of redundancy rather than metabolic compartmentalisation alone.
We thank the reviewer for this suggestion. We wish to point out that the model of metabolic compartmentalisation relies on more than just the experiments in Figure 2 discussed by the reviewer, and is strengthened by our observations of lactate levels which indicate that lactate is produced in cyst cells and consumed in germ cells, as well as our demonstration that a monocarboxylate transporter is required somatically for germ cell survival (which we have strengthened in response to the point below).
Regarding the point raised, we agree that the increase in germ cell death we observe, although substantial (~1.5-2-fold increase in germ cell death, over a baseline where ~30% of germ cell cysts die in normal development as shown by PMID: 23523076) will not lead to sterility as detected by common lab assays. In these assays, single males are mated with several females, and eggs are usually the limiting factor in measuring fertility. However, it is

possible that these males could be less fertile in physiological conditions where they would compete with other males. We have not conducted such fertility assays as they would require optimisation and a considerable amount of time for what we believe to be limited gains.
The experiment suggested by the reviewer, in which we would knock down glycolytic enzymes in both the cyst and germ cell lineages at the same time, would be difficult to interpret, as we observe different baseline rates of cell death in the two driver lines (e.g. compare the controls in Fig. 2C, 2G and 2F). So we might observe increased cell death in knockdowns than if just the somatic driver (*tj-gal4*) was present, but this could simply be an additive effect of higher baseline death with the germline driver itself rather than of germline-specific knockdown. For this reason, we opted not to conduct this experiment. Nonetheless, we are confident that our data support the conclusion that, in normal conditions, germline glycolysis is dispensable for survival. This is supported by the fact that a) germ cells express low levels of glycolytic enzymes and b) germ cell-specific knockdowns of glycolytic enzymes do not cause increased germ cell death. It is possible that somatic knockdown of glycolysis does not cause death of ALL germ cells because of compensatory mechanisms, as suggested by the reviewer. However, firstly, these compensation mechanisms may occur within the cyst cells themselves, or as a result of alternative substrate usage within the germline, rather than upregulation of glycolysis. We have included a discussion of these possibilities on l.339-45. Secondly, the possibility of compensatory mechanisms does not alter our conclusion that, in normal homeostasis, metabolism is compartmentalised across the two cell lineages such that somatic glycolysis supports germ cell survival.

-Related to Figure 4: The identification of CG8034 as the monocarboxylate transporter responsible for lactate transport is currently supported by a single RNAi line, which is insufficient. Additional experiments could include: (1) generating a clean CRISPR mutant, which is relatively straightforward; (2) performing a CRISPR-mediated somatic knockout using a gRNA line available from a stock center; and (3) conducting a rescue experiment by overexpressing an RNAi- or gRNA-resistant, codon-optimised form of CG8034.
We thank the reviewer for raising this point and for giving us the opportunity to strengthen our manuscript. We agree that a single RNAi was not ideal; we had attempted to use a second line (VDRC#106660) which gave no phenotype. However we were confident in our conclusions as we used a lactate reporter to show that knockdown of CG8034 in cyst cells resulted in accumulation of lactate in cyst cells and decreased lactate in germ cells, supporting its role in lactate transport.
We had in fact designed a CRISPR mutant for this gene before submitting the manuscript, but this was not quite as straightforward as suggested and we only obtained a mutant line recently. *CG8034/mlm* is located on the X chromosome, meaning that we had to design a conditional allele in case a mutant was hemizygous lethal. We are pleased to include these data in the revised manuscript in Figures 4 and S5, and described in the text on l. 261-73. The allele is excised by expression of Flp, and we show that expression of Flp with *tj-Gal4* (Fig. 4M) or *nos-Gal4* (Fig. 4N) results in increased germ cell death compared to flies carrying the allele and driver but no Flp. These results phenocopy the knockdown, strengthening our conclusions. In addition, we also conducted the somatic CRISPR experiment suggested by the reviewer. This is shown in Fig. S5E and shows a similar increase in germ cell death.
In sum, we believe that addressing this point has improved this manuscript and strengthened our conclusions as to the role of *CG8034/mlm*.

-Related to Figure 5: In this final figure, the authors use genetic manipulations of Pdk and Pdha1 in cyst cells to modulate oxidative phosphorylation and the pool of pyruvate/lactate available for germ cells, thereby demonstrating metabolic compartmentalisation between

somatic and germ cells. These experiments are very interesting but would be strengthened by rescue experiments. Specifically, the increased cell death observed upon Pdk knockdown should be rescued by simultaneous Mpc1 knockdown (as Mpc1 knockdown alone has no phenotype). Similarly, the decreased cell death observed upon Pdha1 knockdown should also be rescued by concurrent Mpc1 knockdown.
We thank the reviewer for their suggestion. Unfortunately, as pointed out by the reviewer, knockdown of Mpc1 has no phenotype. However, the decreased germ cell death observed upon knockdown of Pdha1 indicates that at least some pyruvate is imported into cyst cell mitochondria. This suggests that other mitochondrial transporters may be responsible for importing pyruvate into cyst cell mitochondria or that we do not achieve effective knockdown with the reagents we have used. Given this, the rescue experiments would be very hard to interpret and would not substantially strengthen our argument. While there may be many ways to promote germ cell death, we believe that observing *reduced* germ cell death upon knockdown of Pdha1 in the soma is one of the best arguments in favour of somatic pyruvate being a limiting resource for the germline.

Minor comments
-Related to Figure 2: The levels of germline cell death appear inconsistent across panels C, G and H. In panel C, the control condition shows around five dying cysts per testis (increasing to 7-8 under experimental conditions), whereas in panels G and H the control condition already reaches 7-8 cysts and no further effect is observed in the experimental conditions. These discrepancies may stem from differences in genetic background. However, since the apparent phenotype overlaps with the control condition in some panels, additional controls are needed to substantiate the findings. At present, only a single control condition is shown. Both parental controls-Gal4 alone and RNAi alone-should be included in all three graphs, and it should be clearly indicated which control is displayed.
We apologise for not labelling our graphs clearly. All the controls refer to the appropriate Gal4 driver crossed to a control strain (*y,w; ;*), such that the progeny are collected and processed together with those of the experimental crosses. We have updated the methods section (l.556-7) to indicate this. As the reviewer noted, different genotypes display different levels of germ cell death, with higher levels in the *nos-gal4* and *bam-gal4* background. This may be because expressing Gal4 in the germline causes some death, or because of other loci in the genetic background. We note that we can still detect increases in germ cell death in these genotypes (e.g. Fig. 3Q).
We have also counted Lysotracker-positive cysts to measure germ cell death in each of the parental RNAi strains we use to knock down glycolytic enzymes. These results are now shown in Fig. S2B of the revised manuscript. All but one of the RNAi lines show lower germ cell death than a wild type strain and than the lowest control (*tj-Gal4)*. Thus, the increased germ cell death we observe upon somatic knockdown of glycolytic enzymes is not due to genetic background.

-Related to Figure 2: Although not essential, it would be informative to test whether sugar starvation exacerbates the phenotype. Showing an increased dependence of the germline on somatic glycolytic activity under nutrient stress could further strengthen the paper.
We agree with the reviewer that how diet influences male gamete production and whether dietary sugar levels influence somatic glycolysis are interesting questions. However, these lie beyond the scope of this manuscript as answering them would require substantial characterisation, as it is likely that circulating trehalose levels are buffered to some extent by the fat body and it would be important to determine that any phenotype caused by dietary manipulation is due to changes in somatic glycolytic flux, and not indirectly by changes in

other tissues. These experiments do indeed form part of our future plans in the lab, and we look forward to seeing how this work develops.

-Related to Figure 2: To rule out an indirect effect mediated by somatic cell death, the author quantifies Zfh1-positive cells. Zfh1 marks CySCs and their immediate daughters, while Eya labels post-mitotic, differentiated cyst cells. In this context, it would also be important to quantify Eya-positive cells in the Tret1 knockdown, for example, to confirm that terminal differentiation of the somatic lineage is not impaired
As suggested by the reviewer, we have counted Eya-positive cells in the Tret1 knockdown. These results are presented in Fig. S2E and in the text on l.159-60. We observe no change in the number of Eya-positive cells, further strengthening our conclusion that glycolysis does not influence cyst cell differentiation or survival.

Referee #3:

This manuscript from the Amoyel lab asks a fundamentally important question about how somatic cells provide nutrients to germ cells, which are isolated through encapsulation by somatic cells. This is an important question that everyone should have wondered, but nobody has. By a series of elegant and convincing experiments, the paper convincingly demonstrates how somatic cells provide the product of glycolysis to the germ cells. Studying metabolism in a manner that maintains cell-type-specific resolution is extremely difficult. But this study achieved this at an unprecedented level (using imaging and genetics), and contains 3 papers-worth conclusions. This is a fundamentally important paper, and I enthusiastically support publication of this paper.
We thank the reviewer for their extremely positive appraisal of our work!

Minor comments:
-Fig2: when glycolysis enzymes are inhibited in somatic cells, germ cell death was upregulated without changing somatic cell composition. But this is a very important point (germ cell death is not due to death of somatic cells), so it would be great if they can provide somewhat more quantitative data to support this point.
We agree that it is important to show that non-cell autonomous germ cell death upon cyst cell glycolytic knockdown is not a consequence of autonomous death. In the manuscript, we have shown a quantification of Dcp-1 positive dying cyst cells in each of the glycolytic knockdowns (Fig. S2D). We could not detect any increase in cyst cell death, while germ cell death is increased (Fig. 2C). To further confirm this, and in response to a comment by reviewer 2 above, we counted Eya-positive cyst cells in Tret1 knockdowns and observed no difference (Fig. S2E). Since we could detect no increase in cell death, no change in CySC numbers (Fig. S2C) and no change in cyst cell numbers, we conclude that somatic cell death is not increased. We wanted to also add an experiment testing for a functional role of cell death in cyst cells. In the original manuscript, we presented data in which we expressed the apoptosis inhibitor Diap1 in cyst cells together with Ldh RNAi (Fig. S4D). Ldh knockdown in cyst cells induced germ cell death, but blocking cyst cell death did not affect the increase in germ cell death. We attempted to reproduce this experiment with a

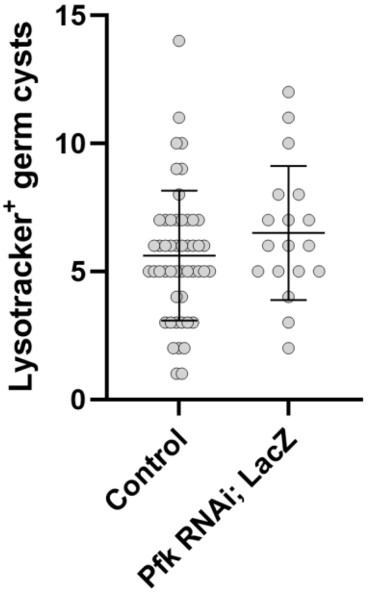

glycolytic enzyme in addition to Ldh, which is the last step in the pathway. However, as indicated in the graph on the right, when expressing Pfk RNAi in cyst cells together with a LacZ titration control, there was no longer a significant increase in germ cell death. This indicates that the RNAi is sensitive to expression levels, precluding us from determining whether co-expression of Diap1 could alter germ cell death.
In sum, although we could not show an additional functional experiment targeting a glycolytic enzyme specifically, our data show that cyst cell death does not increase and that cyst cell numbers do not change upon knockdown of glycolysis, and that preventing cyst cell death together with knockdown of Ldh does not rescue germ cell death. These results together indicate strongly that cyst cell death upon knockdown of the glycolytic pathway does not precede germ cell death.

-they convincingly show that glycolytic enzymes (e.g. pyk) are not required in germline. But any speculation why it is expressed in germline if not necessary? (no need to even include in the revised text, if it is disruptive to the flow).
We thank the reviewer for raising this point which we had not considered. We have added a paragraph in the discussion (l.368-80) mentioning possible reasons why germ cells express glycolytic enzymes.

1st Revision - Editorial Decision **22nd Apr 2026**

Dear Marc,

Thank you for submitting a revised version of your manuscript to The EMBO journal. I sincerely apologise for the protracted assessment of your manuscript due to the high number of manuscripts that we currently receive. Your study has now been seen by two of the original referees, who find that their previous concerns have been sufficiently addressed and now recommend publication of the manuscript after minor revisions.

In addition to the remaining reviewer requests, there are a few editorial points that need to be addressed before I can extend the official acceptance of the manuscript:

1. Please note that it is The EMBO Journal policy for the transcript of the editorial process (containing referee reports and your response letters) to be published as an online supplement to each paper. If you should prefer removal of any referee-only figures included in the point-by-point response(s), e.g. because they may still be used for future publication or because they have been reproduced from published work by others, please do let us know immediately via response email.
More information is available here: https://www.embopress.org/transparent-process#Review_Process
2. Please submit a complete author checklist, which you can download from our author guidelines (https://media.springernature.com/original/springer-cms/rest/v1/content/27825796/data/v1). Please insert information in the checklist that is also reflected in the manuscript. The completed author checklist will also be part of the Review Process File.
3. The headings and order of the sections in the manuscript text should be as follows: Abstract / Introduction / Results / Discussion / Methods / Data Availability / Acknowledgements / Disclosure and Competing Interests Statement / References / Figure Legends / Expanded View Figure Legends.
4. Please remove the figures from the manuscript text, leaving their legends at the end of the manuscript text file.
5. CRediT has replaced the traditional author contributions section because it offers a systematic, machine-readable author contributions format that allows for more effective research assessment. Please remove the Authors Contributions from the manuscript and use the free text boxes beneath each contributing author's name in our online submission system to add specific details on the author's contribution. More information is available in our guide to authors.
6. Please rename "Competing interests" section into "Disclosure and competing interests statement".
7. Please remove the "Funding" subheading in the "Acknowledgements" section.
8. Please rename Table S1 into "Table EV1" and update the nomenclature accordingly throughout the manuscript and the files.
9. The following figure panels should be mentioned in figure legends:
- Figure 1: C', D', E'
- Figure 3: J', K', N', O'
- Figure EV3: C',D'
10. Figure panels 1C', D', E' and 4N are not mentioned in the manuscript text, please check and correct.
11. Our data editors have flagged the following issues in figure legends that need correcting:
- Please provide the exact p values in the legends of figures 1G, 2C,G,H; 3D,E,I,L,M,P,Q; 4A,D,G,H,I,L,M,N; 5A,D,E; EV2B, EV4D, EV5E.
- Please indicate the statistical test used for data analysis in the legend of figure EV1 F.
- Please provide information on the number and nature of replicates in the legends of figures 1G, EV1 F.
- Please define the error bars in the legends of figures 1G, 2C,G,H; 3D,E,I,L,M,P,Q; 4A,D,G,H,I,L,M,N; 5A,D,E; EV2B,C,D,E; EV4 A-D; EV5E.
- Please define the white asterisks in the legend of figures EV3A-D, EV6A .
- Please define the dashed borders in the legend of figure 1C-E'; 2A,B,D,E; 3F-H, H-O'; 4J, K; EV3A,B.
12. Papers published in The EMBO Journal are accompanied online by a 'Synopsis' to enhance discoverability of the manuscript. It consists of A) a short (1-2 sentences) summary of the findings and their significance, B) 3-4 bullet points highlighting key results (the highlights currently included in the manuscript can be used here) and C) a synopsis image that is 550x300 pixels large (width x height, jpeg or png format). You can either show a model or key data in the synopsis image. Please note that the image size is rather small and that text needs to be readable at the final size.

Please let me know if you have any questions regarding any of these points. You can use the link below to upload the revised files.

Thank you again for giving us the chance to consider your manuscript for The EMBO Journal. I look forward to receiving the final version.

With best wishes,

Ieva

Ieva Gailite, PhD

Senior Scientific Editor
The EMBO Journal
Meyerhofstrasse 1
D-69117 Heidelberg
Tel: +4962218891309
i.gailite@embojournal.org

We realize that it is difficult to revise to a specific deadline. In the interest of protecting the conceptual advance provided by the work, we recommend a revision within 3 months (21st Jul 2026). Please discuss the revision progress ahead of this time with the editor if you require more time to complete the revisions. Use the link below to submit your revision:

https://emboj.msubmit.net/cgi-bin/main.plex

Referee #1:

The authors have addressed all my original concerns.

I have one remaining point (which I had missed in the original review, apologies for that) related to the use of PyronicSF and CanlonicSF sensors. Based on the data, I am interpreting that the sensor fluorescence was measured from fixed tissues. However, I do not find specific information regarding this in the Materials and methods section. In the original references provided (Aburto et al., 2022 & Arce-Molina et al., 2020), it appears that the sensors were imaged in live tissues (I can only access the abstract of Aburto et al).

As the authors of this study tested the sensors with proper controls, I do not question the validity of their data. However, prior to publication, it would be good to clarify this issue and to make sure that the Materials and methods section includes the description of how the tissues were prepared for the imaging of the metabolite sensors.

Congratulations to the authors for an excellent study!

Referee #3:

The authors have addressed my (and other reviewers') concerns fully. I support the publication of this excellent paper that will have a lasting impact in the (broad) field.

We thank the reviewers and editorial team for their careful evaluation of our paper. We have made the following changes:

Reviewer comment:
I have one remaining point (which I had missed in the original review, apologies for that) related to the use of PyronicSF and CanlonicSF sensors. Based on the data, I am interpreting that the sensor fluorescence was measured from fixed tissues. However, I do not find specific information regarding this in the Materials and methods section. In the original references provided (Aburto et al., 2022 & Arce-Molina et al., 2020), it appears that the sensors were imaged in live tissues (I can only access the abstract of Aburto et al).

As the authors of this study tested the sensors with proper controls, I do not question the validity of their data. However, prior to publication, it would be good to clarify this issue and to make sure that the Materials and methods section includes the description of how the tissues were prepared for the imaging of the metabolite sensors.

We have included a sentence in the Materials and methods (l.491-4) explaining that we did image the reporters in fixed tissue. This is a point that has been raised by many colleagues and will be of interest to many labs using these tools as the ability to use them in fixed tissue makes them more widely usable.

2nd Revision - Editorial Decision 30th Apr 2026

Dear Marc,

Thank you for incorporating the final editorial requests in your manuscript. I am now pleased to inform you that your manuscript has been accepted for publication. Congratulations with a nice study!

Before we forward your manuscript to the publishers, I will look into the synopsis text that you kindly provided and will let you know at the beginning of the next week if any edits to the journal style are needed.

Your manuscript will be processed for publication by EMBO Press. It will be copy edited and you will receive page proofs prior to publication. Please note that you will be contacted by Springer Nature Author Services to complete licensing and payment information.

You may qualify for financial assistance for your publication charges - either via a Springer Nature fully open access agreement or an EMBO initiative. Check your eligibility: https://link.springer.com/journal/44318/how-to-publish-with-us

Should you be planning a Press Release on your article, please get in contact with embo_production@springernature.com as early as possible in order to coordinate publication and release dates.

If you have any questions, please do not hesitate to contact the Editorial Office or me directly. Thank you for this exciting contribution to The EMBO Journal!

Best wishes,

Ieva

Ieva Gailite, PhD
Senior Scientific Editor
The EMBO Journal
Meyerhofstrasse 1
D-69117 Heidelberg
Tel: +4962218891309
i.gailite@embojournal.org

*****
Please note that it is The EMBO Journal policy for the transcript of the editorial process (containing referee reports and your response letters) to be published as an online supplement to each paper. If you should prefer removal of any referee-only figures included in the point-by-point response(s), e.g. because they may still be used for future publication or because they have been reproduced from published work by others, please do let us know immediately via response email.
More information is available here: https://link.springer.com/partners/embo-press/editorial-policies#Peer%20review

** Click here to be directed to your login page: https://emboj.msubmit.net

EMBO Press Author Checklist

Corresponding Author Name: Marc Amoyel	
Journal Submitted to: The EMBO Journal	
Manuscript Number: EMBOJ-2025-122434R	

USEFUL LINKS FOR COMPLETING THIS FORM
The EMBO Journal - Author Guidelines
EMBO Reports - Author Guidelines
Molecular Systems Biology - Author Guidelines
EMBO Molecular Medicine - Author Guidelines

Reporting Checklist for Life Science Articles (updated January
This checklist is adapted from Materials Design Analysis Reporting (MDAR) Checklist for Authors. MDAR establishes a minimum set of requirements in transparent reporting in the life sciences (see Statement of Task: 10.31222/osf.io/9sm4x). Please follow the journal's guidelines in preparing your
Please note that a copy of this checklist will be published alongside your article.

Abridged guidelines for figures
1. Data
The data shown in figures should satisfy the following conditions:
- the data were obtained and processed according to the field's best practice and are presented to reflect the results of the experiments in an accurate and unbiased manner.
- ideally, figure panels should include only measurements that are directly comparable to each other and obtained with the same assay.
- plots include clearly labeled error bars for independent experiments and sample sizes. Unless justified, error bars should not be shown for technical
- if n<5, the individual data points from each experiment should be plotted. Any statistical test employed should be justified.
- Source Data should be included to report the data underlying figures according to the guidelines set out in the authorship guidelines on Data

2. Captions
Each figure caption should contain the following information, for each panel where they are relevant:
- a specification of the experimental system investigated (eg cell line, species name).
- the assay(s) and method(s) used to carry out the reported observations and measurements.
- an explicit mention of the biological and chemical entity(ies) that are being measured.
- an explicit mention of the biological and chemical entity(ies) that are altered/varied/perturbed in a controlled manner.
- the exact sample size (n) for each experimental group/condition, given as a number, not a range;
- a description of the sample collection allowing the reader to understand whether the samples represent technical or biological replicates (including how many animals, litters, cultures, etc.).
- a statement of how many times the experiment shown was independently replicated in the laboratory.
- definitions of statistical methods and measures:
  - common tests, such as t-test (please specify whether paired vs. unpaired), simple χ2 tests, Wilcoxon and Mann-Whitney tests, can be unambiguously identified by name only, but more complex techniques should be described in the methods section;
  - are tests one-sided or two-sided?
  - are there adjustments for multiple comparisons?
  - exact statistical test results, e.g., P values = x but not P values < x;
  - definition of 'center values' as median or average;
  - definition of error bars as s.d. or s.e.m.

Please complete ALL of the questions below.
Select "Not Applicable" only when the requested information is not relevant for your study.

Materials

Newly Created Materials	Information included in the manuscript?	In which section is the information available? (Reagents and Tools Table, Materials and Methods, Figures, Data Availability Section)
New materials and reagents need to be available; do any restrictions apply?	Yes	Data availability

Antibodies	Information included in the manuscript?	In which section is the information available? (Reagents and Tools Table, Materials and Methods, Figures, Data Availability Section)
For **antibodies** provide the following information:<br>- Commercial antibodies: RRID (if possible) or supplier name, catalogue number and or/clone number<br>- Non-commercial: RRID or citation	Yes	Reagents and tools table

DNA and RNA sequences	Information included in the manuscript?	In which section is the information available? (Reagents and Tools Table, Materials and Methods, Figures, Data Availability Section)
**Short novel DNA or RNA including primers, probes**: provide the sequences.	Yes	Reagents and tools table, Materials and methods

Cell materials	Information included in the manuscript?	In which section is the information available? (Reagents and Tools Table, Materials and Methods, Figures, Data Availability Section)
**Cell lines:** Provide species information, strain. Provide accession number in repository **OR** supplier name, catalog number, clone number, and/**OR** RRID.	Not Applicable	
**Primary cultures:** Provide species, strain, sex of origin, genetic modification status.	Not Applicable	
Report if the cell lines were recently **authenticated** (e.g., by STR profiling) and tested for mycoplasma contamination.	Not Applicable	

Experimental animals	Information included in the manuscript?	In which section is the information available? (Reagents and Tools Table, Materials and Methods, Figures, Data Availability Section)
**Laboratory animals or Model organisms:** Provide species, strain, sex, age, genetic modification status. Provide accession number in repository **OR** supplier name, catalog number, clone number, **OR** RRID.	Yes	Reagents and Tools table, materials and methods
**Animal observed in or captured from the field:** Provide species, sex, and age where possible.	Not Applicable	
Please detail **housing and husbandry conditions**.	Yes	Materials and methods

Plants and microbes	Information included in the manuscript?	In which section is the information available? (Reagents and Tools Table, Materials and Methods, Figures, Data Availability Section)
**Plants:** provide species and strain, ecotype and cultivar where relevant, unique accession number if available, and source (including location for collected wild specimens).	Not Applicable	
**Microbes:** provide species and strain, unique accession number if available, and source.	Not Applicable	

Human research participants	Information included in the manuscript?	In which section is the information available? (Reagents and Tools Table, Materials and Methods, Figures, Data Availability Section)
If collected and within the bounds of privacy constraints report on age, sex and gender or ethnicity for all study participants.	Not Applicable	

Core facilities	Information included in the manuscript?	In which section is the information available? (Reagents and Tools Table, Materials and Methods, Figures, Data Availability Section)
If your work benefited from core facilities, was their service mentioned in the acknowledgments section?	Yes	Acknowledgements

Design

Study protocol	Information included in the manuscript?	In which section is the information available? (Reagents and Tools Table, Materials and Methods, Figures, Data Availability Section)
If study protocol has been **pre-registered, provide DOI in the manuscript**. For clinical trials, provide the trial registration number **OR** cite DOI.	Not Applicable	
Report the **clinical trial registration number** (at ClinicalTrials.gov or equivalent), where applicable.	Not Applicable	

Laboratory protocol	Information included in the manuscript?	In which section is the information available? (Reagents and Tools Table, Materials and Methods, Figures, Data Availability Section)
Provide DOI OR other citation details if **external detailed step-by-step protocols** are available.	Not Applicable	

Experimental study design and statistics	Information included in the manuscript?	In which section is the information available? (Reagents and Tools Table, Materials and Methods, Figures, Data Availability Section)
Include a statement about **sample size** estimate even if no statistical methods were used.	Yes	Figures
Were any steps taken to minimize the effects of subjective bias when allocating animals/samples to treatment (e.g. **randomization procedure**)? If yes, have they been described?	Not Applicable	
Include a statement about **blinding** even if no blinding was done.	Not Applicable	
Describe **inclusion/exclusion criteria** if samples or animals were excluded from the analysis. Were the criteria pre-established?<br><br>If sample or data points were omitted from analysis, report if this was due to attrition or intentional exclusion and provide justification.	Not Applicable	
For every figure, are **statistical tests** justified as appropriate? Do the data meet the assumptions of the tests (e.g., normal distribution)? Describe any methods used to assess it. Is there an estimate of variation within each group of data? Is the variance similar between the groups that are being statistically compared?	Yes	Materials and methods, Figure legends

Sample definition and in-laboratory replication	Information included in the manuscript?	In which section is the information available? (Reagents and Tools Table, Materials and Methods, Figures, Data Availability Section)
In the figure legends: state number of times the experiment was **replicated** in laboratory.	Yes	Figures
In the figure legends: define whether data describe **technical or biological replicates**.	Yes	Figure legends

Ethics

Ethics	Information included in the manuscript?	In which section is the information available? (Reagents and Tools Table, Materials and Methods, Figures, Data Availability Section)
Studies involving **human participants**: State details of **authority granting ethics approval** (IRB or equivalent committee(s), provide reference number for approval.	Not Applicable	
Studies involving **human participants**: Include a statement confirming that **informed consent** was obtained from all subjects and that the experiments conformed to the principles set out in the WMA Declaration of Helsinki and the Department of Health and Human Services Belmont Report.	Not Applicable	
Studies involving **human participants:** For publication of **patient photos**, include a statement confirming that consent to publish was obtained.	Not Applicable	
Studies involving experimental **animals**: State details of **authority granting ethics approval** (IRB or equivalent committee(s), provide reference number for approval. Include a statement of compliance with ethical regulations.	Not Applicable	
Studies involving **specimen and field samples:** State if relevant **permits** obtained, provide details of authority approving study; if none were required, explain why.	Not Applicable	

Dual Use Research of Concern (DURC)	Information included in the manuscript?	In which section is the information available? (Reagents and Tools Table, Materials and Methods, Figures, Data Availability Section)
Could your study fall under dual use research restrictions? Please check biosecurity documents and list of **select agents and toxins** (CDC): https://www.selectagents.gov/sat/list.htm	Not Applicable	
If you used a select agent, is the security level of the lab appropriate and reported in the manuscript?	Not Applicable	
If a study is subject to dual use research of concern regulations, is the name of the **authority granting approval and reference number** for the regulatory approval provided in the manuscript?	Not Applicable	

Reporting

The MDAR framework recommends adoption of discipline-specific guidelines, established and endorsed through community initiatives. Journals have their own policy about requiring specific guidelines and recommendations to complement MDAR.

Adherence to community standards	Information included in the manuscript?	In which section is the information available? (Reagents and Tools Table, Materials and Methods, Figures, Data Availability Section)
State if relevant guidelines or checklists (e.g., **ICMJE, MIBBI, ARRIVE, PRISMA**) have been followed or provided.	Not Applicable	
For **tumor marker prognostic studies,** we recommend that you follow the **REMARK** reporting guidelines (see link list at top right). See author guidelines, under 'Reporting Guidelines'. Please confirm you have followed these guidelines.	Not Applicable	
For **phase II and III randomized controlled trials**, please refer to the **CONSORT** flow diagram (see link list at top right) and submit the CONSORT checklist (see link list at top right) with your submission. See author guidelines, under 'Reporting Guidelines'. Please confirm you have submitted this list.	Not Applicable	

Data Availability

Data availability	Information included in the manuscript?	In which section is the information available? (Reagents and Tools Table, Materials and Methods, Figures, Data Availability Section)
Have **primary datasets** been deposited according to the journal's guidelines (see 'Data Deposition' section) and the respective accession numbers provided in the Data Availability Section?	Yes	Data availability section
Were **human clinical and genomic datasets** deposited in a public access-controlled repository in accordance to ethical obligations to the patients and to the applicable consent agreement?	Not Applicable	
Are **computational models** that are central and integral to a study available without restrictions in a machine-readable form? Were the relevant accession numbers or links provided?	Not Applicable	
If publicly available data were reused, provide the respective **data citations in the reference list**.	Yes	References cited in the text and included in the reference list.
